# Insights into the regulatory role of RNA methylation modifications in glioma

**DOI:** 10.1186/s12967-023-04653-y

**Published:** 2023-11-14

**Authors:** Shengrong Long, Yu Yan, Hongyu Xu, Lesheng Wang, Jiazhi Jiang, Ziyue Xu, Runming Liu, Qiangqiang Zhou, Xiaopeng Huang, Jincao Chen, Zhiqiang Li, Wei Wei, Xiang Li

**Affiliations:** 1 Department of Neurosurgery, Zhongnan Hospital of Wuhan University, Wuhan, 430071 China; 2 Brain Research Center, Zhongnan Hospital of Wuhan University, Wuhan, 430071 China

**Keywords:** RNA methylation modification, Glioma, Epitranscriptome, m6A, m5C

## Abstract

Epitranscriptomic abnormalities, which are highly prevalent in primary central nervous system malignancies, have been identified as crucial contributors to the development and progression of gliomas. RNA epitranscriptomic modifications, particularly the reversible modification methylation, have been observed throughout the RNA cycle. Epitranscriptomic modifications, which regulate RNA transcription and translation, have profound biological implications. These modifications are associated with the development of several cancer types. Notably, three main protein types—writers, erasers, and readers, in conjunction with other related proteins, mediate these epitranscriptomic changes. This review primarily focuses on the role of recently identified RNA methylation modifications in gliomas, such as N6-methyladenosine (m6A), 5-methylcytosine (m5C), N7-methylguanosine (m7G), and N1-methyladenosine (m1A). We delved into their corresponding writers, erasers, readers, and related binding proteins to propose new approaches and prognostic indicators for patients with glioma.

## Introduction

Glioma is a widely prevalent primary malignant tumor of the central nervous system, comprising up to 77% of all primary brain malignancies [1, 2], and is prone to recurrence with a poor prognosis. According to the World Health Organization (WHO) classification, grade 4 glioblastoma multiforme (GBM) is the worst malignancy of this type [3]. The median survival time for patients diagnosed with GBM is less than 15 months, with only a small percentage of patients (3–5%) surviving beyond 3 years [4], even with the standard treatment approach of surgical resection followed by concurrent chemoradiotherapy. Given these statistics, identifying potential diagnostic and therapeutic targets by exploring the origins of gliomas has been the primary focus of glioma research, primarily because of the high recurrence rate and poor prognosis associated with this malignancy. Despite extensive research, the pathogenesis and molecular characteristics of gliomas remain unclear.

Recent studies demonstrated the crucial role of epitranscriptomic modifications in regulating tumor occurrence and development [5]. This regulatory mechanism plays significant roles in cell fate, proliferation, metabolism, and pathological processes. Given their importance, a deeper understanding of the role of epitranscriptomic modifications in the pathogenesis of tumors, including gliomas, is critical for the development of novel diagnostic and therapeutic approaches [6–9].

Epigenetics is a complex process involving various modifications such as histone modification, chromatin remodeling, nucleosome localization, DNA methylation, and RNA modification. In particular, RNA modifications encompass over 170 covalent modifications, mostly methylation modifications, including m6A, m5C, and m7G methylation. Thus, a comprehensive understanding of various epitranscriptomic modifications and their roles in glioma pathogenesis is crucial for developing novel and effective diagnostic and therapeutic strategies for different diseases [10]. Methylation is a crucial epigenetic modification that regulates gene expression, RNA stability, and the nuclear export or import of nucleic acids and proteins. Specifically, nucleoside methyltransferases catalyze RNA methylation, thereby playing a crucial role in epitranscriptomic regulation.

In this review, we present an overview of recent research progress on several common RNA methylation modifications and their corresponding regulatory enzymes in gliomas. Hence, this review aims to discuss the potential research directions for the study of RNA modification and its potential role in the origin, diagnosis, and treatment of glioma to improve patient outcomes and develop more effective therapies.

**Fig. 1:**
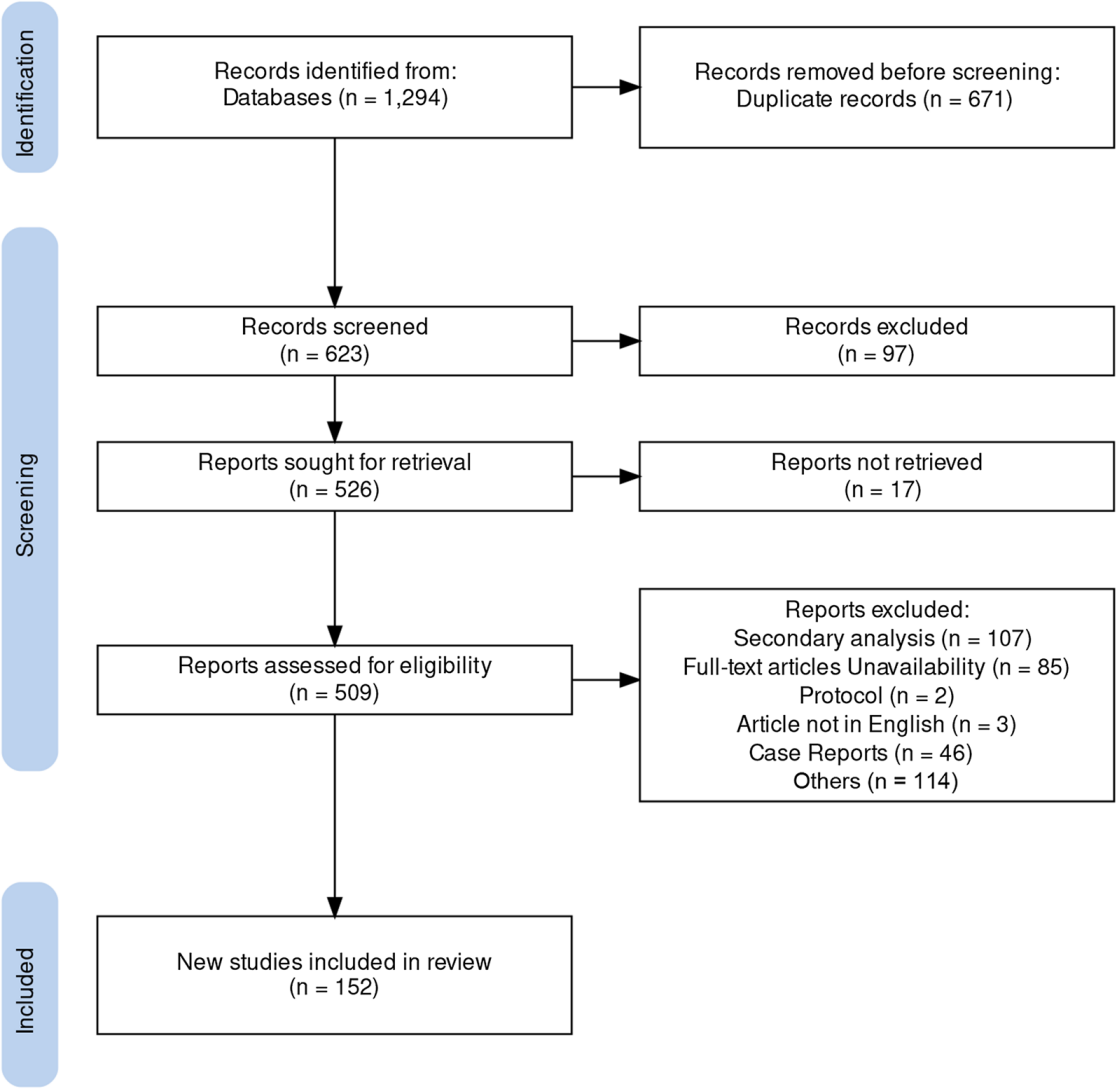
Review flow diagram

**Fig. 2:**
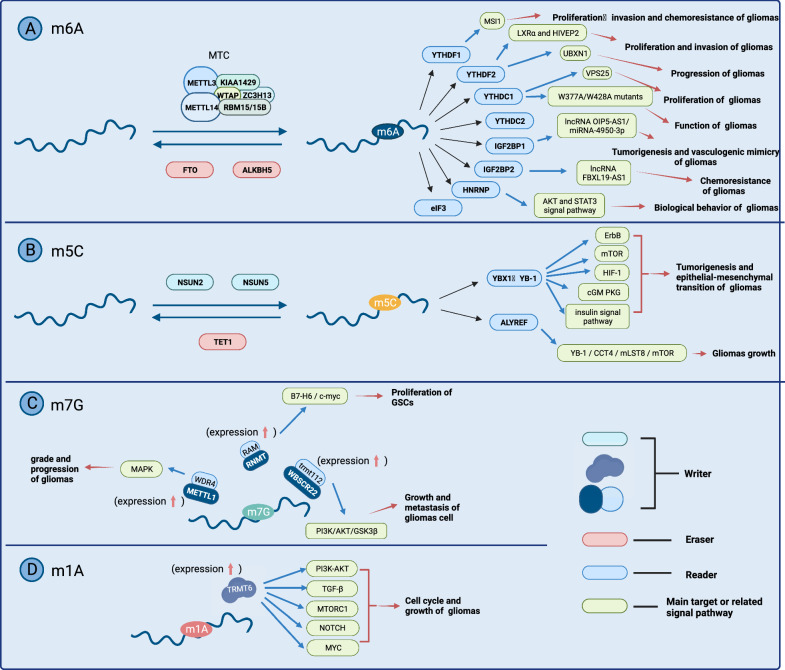
RNA modification plays essential roles in pathological processes associated with gliomas. **A** Functions of m6A modification in gliomas and its writers, erasers, and readers, which maintain the dynamic balance of m6A modifications. **B** Functions of m5C modification in gliomas. **C** m7G. **D** m1A

The flowchart of this review (Fig. 1). A comprehensive understanding of the role of RNA methylation in glioma pathogenesis is critical for the developing of novel diagnostic and therapeutic approaches (Table 1) (Fig. 2).

**Table 1:** The roles of RNA methylation modification in gliomas

Modification regulators	Roles	Expression	Target or related pathway	Related biological characters	Refs
m6A					
Writer					
METTL3	Oncogene	Upregulated	SOX2	Tumor growth	[20]
			MGMT、ANPG	Drug resistance	[21]
			ADAR、APOBEC3A	RNA processing and Cancer-related pathways	[22]
			SRSF	Tumor growth and progression	[24]
			UBXN1	Tumor progression	[66]
	Tumor suppressor	Downregulated	–	Growth and renewal of GSCs	[27]
			circDLC1	Proliferation	[156]
			PI3K/Akt、COL4A1、HSP90	Proliferation、invasion and migration of cancer cell	[28, 29, 30]
METTL14	Tumor suppressor	Upregulated or downregulated	–	Disturbance of oncogene and anti-oncogene expression	[27]
			ASS1	Proliferation、growth、invasion and migration of cancer cell	[31]
WTAP	Oncogene	Upregulated	–	Associated with prognosis	[157, 158]
RBM15/15B	–	Upregulated	–	Related to grade of gliomas and drug resistance	[35, 36]
KIAA1429/VIRMA	–	Upregulated and downregulated in GBMs com- paring with LGGs		Tumor procedure	[159]
ZC3H13	–	Downregulated	–	Drug resistance	[39, 160]
Eraser					
FTO	Oncogene	Upregulated	MYC-miR-155/23a Cluster-MXI1	Drug resistance	[45]
			–	Growth of GSC	[27]
			PDK1	Aerobic glycolysis of cancer cell	[46]
	Tumor suppressor	Downregulated	FOXO3a	Poor prognosis and malignant tumor behavior	[48, 49]
ALKBH5	Tumor suppressor	Upregulated	G6PD mRNA	Proliferation and metabolism of cancer cell	[54]
			–	Epithelial-mesenchymal transition and vasculogenic mimicry	[55]
			NANOG	Drug resistance	[56]
			Homologous recombination (CHK1、RAD51)	Resistance to radiation	[57]
			SOX2	Proliferation、apoptosis and drug resistance of cancer cell	[58]
			FOXM1	Proliferation and renewal of cancer cell	[59]
Reader					
YTHDF1	Oncogene	Upregulated	MSI1	Proliferation、invasion and chemoresistance of gliomas	[69]
YTHDF2	Oncogene	Upregulated	LXRα and HIVEP2	Proliferation and invasion of gliomas	[65]
			–	Grade of gliomas and prognosis	[66]
			UBXN1	Progression of gliomas	[66]
YTHDC1	Oncogene	–	VPS25	Proliferation of cancer cell	[161]
			W377A/W428A mutants	Function of cancer cell	[24]
YTHDC2	–	Upregulated and downregulated in GBMs com- paring with LGGs	–	Prognosis of low-grade gliomas	[73]
IGF2BP1	Oncogene	Upregulated	Lnc00689/ miR-526b-3p/IGF2BP1	Tumorigenesis	[72]
IGF2BP2	Oncogene	Upregulated	lncRNA OIP5-AS1/miRNA-4950-3p	Tumorigenesis and vasculogenic mimicry	[77]
			IGF2BP2/lncRNA FBXL19-AS1/ZNF765	Chemoresistance	[78]
HNRNP	Oncogene	Upregulated and downregulated in GBMs com- paring with LGGs	AKT and STAT3 signal pathway	Biological behavior of tumor	[87]
eIF3	Oncogene	Downregulated		Cell cycle and apoptosis	[95]
m5C					
Writer					
NSUN2	–	Upregulated	ATX	Cell cycle and migration	[105]
NSUN5	–	Downregulated	rRNA	stress survival adaptations	[107, 108]
Eraser					
TET1	Tumor suppressor	Upregulated	-	Repair of DNA damage	[120, 162]
Reader					
YBX1 (YB-1)	Oncogene	Upregulated	ErbB, mTOR, HIF-1, cGM PKG, insulin signal pathway	Tumorigenesis and epithelial-mesenchymal transition	[123]
			YB-1 / CCT4 / mLST8 / mTOR	Tumor growth	[163]
ALYREF	–	Upregulated	–	Prognosis factor	[125]
m7G					
Writer					
METTL1/ WDR4	Oncogene	Upregulated	MAPK signal pathway	Related to the grade and progression of gliomas	[134, 138]
RNMT/ RAM	Oncogene	Upregulated	B7-H6/c-myc	Proliferation of GSCs	[141]
WBSCR22/TRMT112	Oncogene	Upregulated	PI3K/AKT/GSK3β signal pathway	Growth and metastasis of gliomas cell	[143]

### M6Am6A methylation

In the early 1970s, Desrosiers et al. [11] identified and characterized a novel RNA epitranscriptomic modification, N6-methyladenosine (m6A), in mRNA extracted from Novikoff hepatoma cells. m6A modification involves the methylation of the N6-position of adenosine on RNA. It is currently recognized as the most prevalent, abundant, and evolutionarily conserved form of internal RNA modification. m6A modification of mRNA is primarily concentrated within a conserved sequence containing the RRACH motif (R: adenine or guanine; A: adenine; C: cytosine; H: adenine, cytosine, or uracil). The m6A modification of RNA is primarily located within the mRNA transcription start site and 3′-untranslated region (UTR), averaging 2–3 m6A modification sites in each transcript [12]. In addition to mRNA, m6A modifications are present in other types of RNA, such as ribosomal RNA (rRNA), transfer RNA (tRNA), small nucleolar RNA, microRNA (miRNA), long noncoding RNA (lncRNA), and circular RNAs (circRNAs). The widespread presence of m6A modifications underscores their potential significance in regulating gene expression and overall cellular function. Therefore, further research is warranted to elucidate the roles of m6A modifications in glioma pathogenesis with the ultimate goal of developing novel diagnostic and therapeutic approaches [13–16].

RNA m6A modifications involve three types of proteins: methyltransferases (writers), demethylases (erasers), and m6A binding proteins (readers) [17]. The sixth adenosine site in RNA can undergo methylation by m6A methyltransferase or demethylation by demethylase. Furthermore, the m6A-binding protein specifically identifies m6A-modified RNA. The interplay among these proteins influences m6A methylation, making it a reversible process. Extensive research indicates that m6A RNA methylation plays an essential role in post-transcriptional gene expression regulation, encompassing RNA splicing, stability, export, and degradation [18]. Under normal physiological conditions, m6A modifications are maintained by a dynamic balance between methyltransferase complexes and demethylases. However, a disruption of this balance can lead to tumorigenesis. During cancer progression, dynamic changes in m6A contribute to rapid tumor adaptation to microenvironmental alterations [15, 19]. Much research has highlighted the role of m6A methylation regulatory factors in gliomas, with some regulatory factors potentially serving as prognostic markers.

### m6A writers

#### METTL3

METTL3, the first identified m6A methyltransferase, contains a gene located on 14q11.2, comprising 10 exons. It modulates the expression of oncogenes and tumor suppressor genes at the post-transcriptional level, including mRNA stability and translation. Visvanathan et al. [20] reported that METTL3 was upregulated in human GBM tissues and induced m6A modification by binding to the 3′-UTR of SOX2 mRNA. METTL3 knockdown inhibited SOX2 expression, enhanced the sensitivity of tumor cells to γ-radiation in vitro, and inhibited the growth of GBM cells in mice, thus playing a carcinogenic role. Shi et al. [21] found that, in glioma, METTL3 promotes drug resistance to temozolomide (TMZ) by increasing the dependence of O6-methylguanine (O6-MeG)-DNA methyltransferase (MGMT) and ANPG on m6A. Visvanathan et al. [22] suggested that METTL3 plays a crucial role in RNA processing by regulating the RNA-editing enzymes ADAR and APOBEC3A to alter adenosine-to-inosine and cytidine-uridine RNA editing. The occurrence of abnormal alternative splicing events increased significantly after METTL3 knockdown. By analyzing the direct and indirect targets of RNA regulation after knocking down METTL3, it was found that METTL3 is essential in NOTCH, NF-κB, Wnt, c-Myc, TGF-β and other key carcinogenesis signaling pathways related to GBM [23]. Li et al. [24] were the first to reveal the mechanism by which m6A modification regulates nonsense-mediated mRNA degradation (NMD) to promote GBM growth and progression. They found that METTL3-mediated m6A modification can affect the expression levels of serine/arginine-rich splicing factors (SRSFs) by upregulating BCL-X or NCOR2 and inhibiting YTHDC1-dependent NMD. METTL3-mediated m6A modification, with the assistance of HuR, enhances the stability of MALAT1 and activates NF-κB, promoting the malignant progression of IDH wild-type glioma [25]. Moreover, the expression of METTL3 was positively correlated with a higher malignancy grade and grim prognosis in IDH-wild-type gliomas but not with IDH-mutant gliomas [26].

**Fig. 3:**
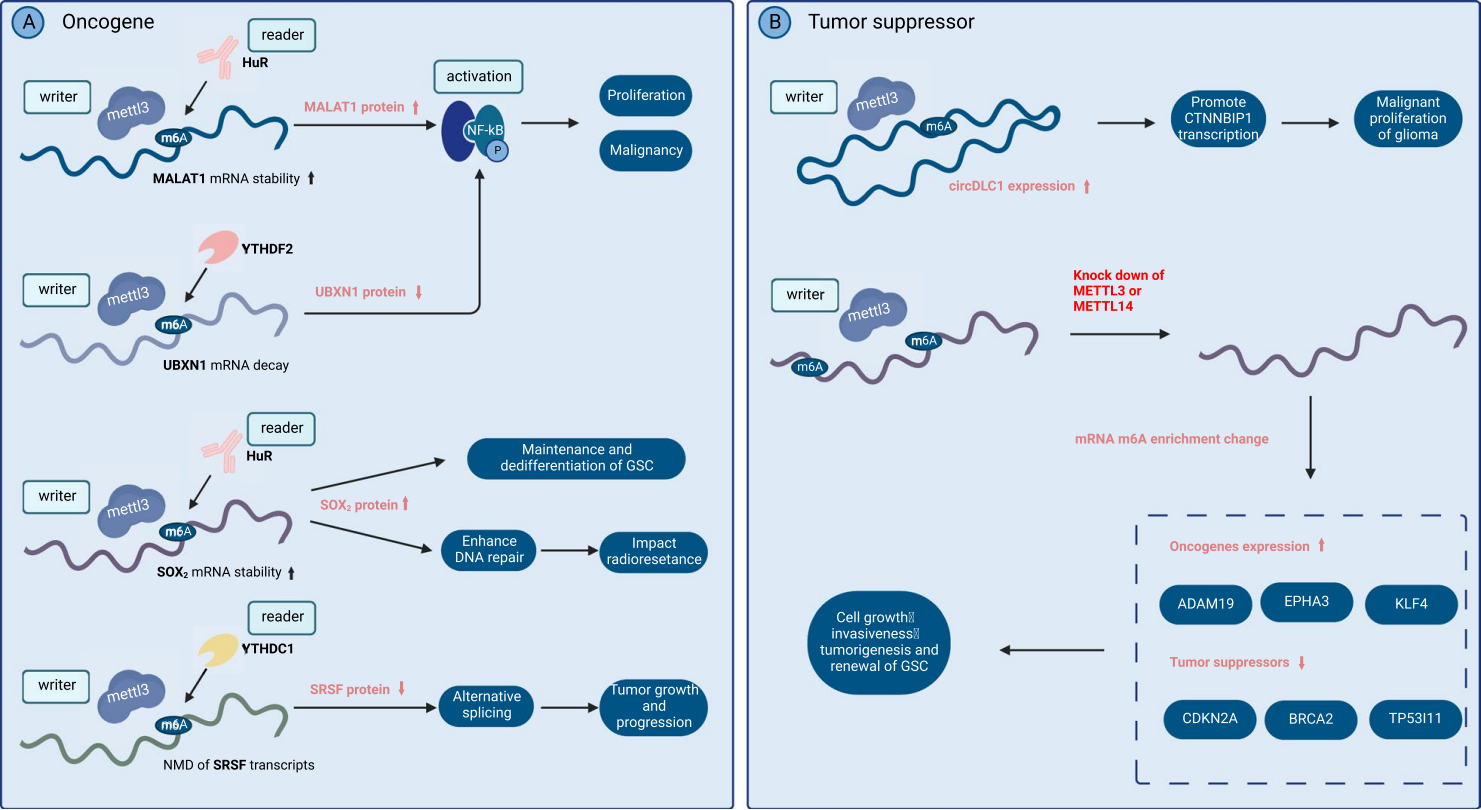
METTL3 plays opposite roles in gliomas because of different target genes and RNA-binding proteins. **A** METTL3 acts as an oncogene and is upregulated in gliomas. MALAT1, UBXN1, SOX2, and SRSF are all important targets of METTL3. **B** METTL3 also acts as a tumor suppressor and is downregulated in gliomas. CircDLC 1 is one of many important targets of METTL3. The knockdown of METTL3 or METTL14 promotes tumorigenesis and malignant tumor behavior

However, some studies have shown that METTL3 and a highly m6A-modified state may suppress tumor development. Inhibiting m6A enrichment via METTL3 knockdown resulted in enhanced growth, self-renewal, and tumor progression of glioma stem-like cells (GSCs), as reported by Cui et al [27]. Ji et al. [28] Han et al. [29] also showed that METTL3 exerts a regulatory function in glioma cell proliferation, migration, and invasion by inhibiting the PI3K/AKT signaling pathway, suggesting that this pathway is a potential therapeutic target for glioma treatment. Li et al. [30] revealed that m6A levels decreased in glioma tissues due to reduced METTL3 and increased FTO expression. The upregulation of m6A has also been shown to reduce migration and proliferation and regulate cell proliferation via HSP90-mediated apoptosis in U251 cells. These conflicting views on the role of METTL3—whether it promotes or suppresses glioma—may be attributed to the diverse target genes affected by m6A and the heterogeneity of tumor stem cells, both genetically and non-genetically (Fig. 3).

#### METTL14

METTL14, located on chromosome 4q26 and comprising 12 exons, shares 43% sequence similarity with METTL3. METTL14 knockdown promotes a malignant phenotype by upregulating the expression of oncogenes (e.g., ADAM19) and downregulating the expression of tumor suppressor genes (e.g., CDKN2A). These findings highlight the critical role of METTL14 in glioma progression and emphasize its potential as a therapeutic target [27]. Miao et al. [31] demonstrated that METTL14 promotes glioma development by inhibiting the expression of argininosuccinate synthase 1 (ASS1) in an m6A-dependent manner and that high ASS1 expression can inhibit the proliferation, migration, invasion, and growth of glioma cells. Concurrently, METTL14 facilitates the m6A modification of ASS1, leading to a reduction in ASS1 mRNA levels. Suppression of ASS1 mRNA expression by METTL14 relies on YTHDF2-mediated m6A modification and degradation. These findings indicate that the METTL14/ASS1/YTHDF2 regulatory axis is a potential therapeutic target for glioma treatment. Interestingly, compared to METTL3, METTL14 demonstrates more pronounced effects and primarily acts as an inhibitor of glioma progression, offering a novel avenue for therapeutic interventions in this disease.

#### WTAP

WTAP, also known as Mum2, interacts with the Wilms’ tumor 1 gene and plays a coordinating role in RNA methylation. Although WTAP lacks catalytic functions, it binds to RNA and recruits METTL3 and METTL14 for methylation [27, 32]. Earlier studies classified WTAP as a tumor suppressor, but Xi et al. found that it was overexpressed in GBM, and its expression was closely related to the glioma grade. In addition, high WTAP expression is correlated with low postoperative survival in patients with glioma and can be used as a prognostic marker [15]. Xi et al. [33] also found that miR-29a binds to the 3′-UTR of QKI-6, inhibiting the expression of WTAP, a downstream target of QKI-6, thereby suppressing the malignant behavior of GSCs. Loss of WTAP curbs cancer cell migration and invasion, potentially through the regulation of EGFR activity [34]. Such observations underscore WTAP's oncogenic significance in gliomas and emphasize its promise as a therapeutic target in glioma management.

#### RBM15/15B

RNA-binding motif protein 15/15B (RBM15/RBM15B), also referred to as OTT or SPEN, is adjacent to the methylated RRACH motif in the mRNA transcriptome and can bind to the METTL3-WTAP complex. It is recruited to a specific site for methylation, so it is also considered a methyltransferase [35]. Chai et al. [36] analyzed the mRNA expression levels of 13 m6A methylation regulators in a clinical cohort of 904 patients with glioma from The Cancer Genome Atlas and Chinese Glioma Genome Atlas databases. They found that WTAP and RBM15 expression positively correlated with glioma grade and that the high-risk group with high RBM15 expression was more sensitive to TMZ. These discoveries accentuate the prospect of devising RBM15/15B-centric therapeutic strategies.

#### KIAA1429/VIRMA

KIAA1429 is a pivotal m6A methyltransferase-related protein and is the most abundant component of the methyltransferase complex (MTC). As a scaffold for coordinating core components on RNA substrates, KIAA1429 mediates m6A methylation on specific sequences near the 3′-UTR and termination codon. The knockdown of KIAA1429 in A549 cells resulted in a significant four-fold reduction in the median peak value of m6A compared to the knockdown of METTL3 or METTL14 alone, indicating its essential role in mammalian methylation [26, 37]. Interestingly, while KIAA1429 is upregulated in low grade glioma (LGG), it is downregulated in GBM compared to normal brain tissues [26].

#### ZC3H13

ZC3H13, also termed KIAA0853 or Xio, was recently identified as a methylation regulator. It regulates m6A methylation by forming a ZC3H13-WTAP-virilizer-hakai complex in the nucleus [38]. Chow et al. [39] demonstrated that the adeno-associated virus (AAV)-CRISPR-mediated upregulation of ZC3H13 in GBM altered the gene expression profile of Rb1 mutants, subsequently enhancing resistance to TMZ.

#### m6A erasers

Two enzymes, FTO and ALKBH5, have been identified to participate in the m6A demethylation process [17, 40]. Both enzymes belong to the ALKB homologous protein family of human Escherichia coli DNA alkylation demethylases, which contain a conserved histidine-aspartic acid histidine (HDH) domain that binds divalent iron ions and a dual arginine (RxxR) motif that binds 2-ketoglutarate and recognizes its RNA substrates. It has been discovered that FTO can mediate the demethylation of both m6A and m6Am, specifically on RNA molecules with a poly-A tail. Furthermore, the preference for demethylation appears to be affected by the location of the molecule within the cell, with differences observed between the nucleus and cytoplasm [41]. The subcellular location of FTO dictates its functional specificity. Nuclear FTO regulates m6A demethylation, whereas cytoplasmic FTO mediates the demethylation of both m6A and m6Am [33, 42]. ALKBH5 specifically demethylates m6A in a physiological sequence context, whereas FTO has no sequence requirements for m6A demethylation [43, 44]. The absence of sequence preference for FTO suggests that m6A may not be the optimal substrate for positioning ALKBH5 as the primary m6A demethylase [33].

#### FTO

FTO, the earliest reported m6A demethylase, provided the first evidence of the reversibility and dynamics of m6A modification [40]. FTO is predominantly associated with glioma carcinogenesis. Xiao et al. [45] found that inhibiting FTO could target the Myc-miR-155/23a Cluster-MXI1 feedback loop, enhancing the antitumor effect of TMZ in glioma. Cui et al. [27] demonstrated that FTO has a pro-carcinogenic effect on GBM. GSC growth can be inhibited by increasing the level of m6A modification in GSC mRNAs using the FTO inhibitor methyl chlorofenamic acid ethyl ester (MA2). In addition to mRNA, the m6A modification of lncRNAs also plays an important role in gliomas. One study showed that the lncRNA JPX could improve the stability of PDK1 transcripts by acting on FTO, promoting GBM aerobic glycolysis, and playing a key role in resistance to TMZ [46]. Researchers have discovered that FTO-04, a competitive inhibitor of FTO, selectively prevents neurosphere formation in patient-derived GSCs without affecting healthy neural stem cell-derived neurospheres. This suggests the potential of FTO-04 as a therapeutic agent for diseases associated with dysregulated m6A modifications [47].

In contrast, various pieces of evidence challenge the notion of cancer-promoting effects of FTO in gliomas. Tao et al. [48] reported reduced FTO expression in gliomas, particularly high-grade gliomas, and associated lower FTO expression with adverse clinical outcomes. The interaction between FTO and FOXO3a can inhibit the malignant behavior of tumors by promoting the nuclear translocation and regulating the expression of FOXO3a. Other studies [49] have found that the SPI1 inhibitor DB2313 can restore endogenous FTO expression and reduce the tumor burden of GBM, indicating that FTO is a promising new prognostic indicator and therapeutic molecular target for GBM. FTO plays both pro- and anti-carcinogenic roles in glioma for the same reasons as P53 [50]. The metabolic function of FTO may differ across glioma developmental stages or tissue subtypes, potentially exerting contrasting effects.

#### ALKBH5

ALKBH5 is a non-heme iron (II)/ketoglutarate-dependent dioxygenase with iron-dependent active expression and is localized in the nucleolar patches and subcellular organelles within the nucleus. ALKBH5 may be involved in the alternative splicing of RNA precursors, and its knockdown can promote mRNA nucleation [51, 52]. Dong et al. [53] found that hypoxia induced ALKBH5 upregulation, thereby upregulating CXCL8/IL8 expression and promoting tumor-associated macrophage recruitment to produce an immunosuppressive tumor microenvironment. Notably, hypoxia-induced changes in the transcriptome are associated with an immunosuppressive microenvironment that facilitates tumor evasion. Liu et al. [54] showed that ALKBH5 catalyzes the demethylation of G6PD mRNA, enhances its stability, and promotes its translation. ALKBH5 plays an important role in glioma cell proliferation and energy metabolism by activating the pentose phosphate pathway. Tao et al. [55] revealed that ALKBH5 reduced RNA m6A methylation levels in GBM, promoting GBM growth, epithelial-mesenchymal transition (EMT), and vasculogenic mimicry (VM). Ding et al. [56] found that the knockdown of circ_0072083 in exosomes blocked ALKBH5-mediated demethylation in glioma cells and reduced NANOG expression, thus modulating TMZ resistance. Kowalski et al. [57] reported that, in GSCs, a high expression of the RNA demethylase ALKBH5 enhances the radiotherapy resistance of tumor cells by regulating homologous recombination, including CHK1 and RAD51 expression. Liu et al. [58] identified elevated levels of the lncRNA SOX2OT in TMZ-resistant cells and recurrent GBM patient samples and that it upregulated SOX2 expression by recruiting ALKBH5 to demethylate SOX2 transcripts, activate the Wnt5a/β-catenin signaling pathway, inhibit apoptosis, and promote cell proliferation and TMZ resistance. Zhang et al. [59] showed that ALKBH5 is upregulated in GSCs and enhances cell self-renewal, proliferation, and tumorigenicity by increasing the FOXM1 expression level, which is a key target gene in patients with GBM. These studies suggest that ALKBH5 exerts pro-cancer effects in gliomas mainly by altering tumor cell immunity, metabolism, drug resistance, and radiotherapy resistance.

#### m6A readers

The m6A reader proteins can be broadly classified into three primary groups, with the first being the YTH protein family, which contains an evolutionarily conserved YTH (YT521-B homolog) structural domain. Insights from an RNA pull-down experiment showed that proteins containing the YTH domain are universal m6A binding agents [12]. YTHDF1 (YTH domain family, member 1), YTHDF2, YTHDF3, YTHDC1 (YTH domain-containing 1), and YTHDC2 are members of the YTH protein family. These YTH domain-containing proteins have a wide range of functions, and their specific functions are related to their ability to bind m6A. YTHDF proteins show identical binding to all m6A sites in mRNAs and mediate the degradation of m6A-mRNAs [60].

Another group of m6A reader proteins comprises insulin-like growth factor mRNA-binding proteins (IGF2BP 1–3). IGF2BP improves the stability and storage of target mRNA in an m6A-dependent manner [61]. The third is the nuclear inhomogeneous nuclear ribonucleoprotein (hnRNP) family, which includes hnRNPC, hnRNPG, and hnRNPA2B1. These hnRNP proteins modulate m6A-containing RNA transcripts and selectively bind to m6A-containing transcripts [62].

#### YTHDF1-3

Among the proteins with YTH domains, YTHDF1, YTHDF2, and YTHDF3 play pivotal roles in gliomas. YTHDF1 and YTHDF3 synergistically enhance the translation efficiency of their target RNA [63], while YTHDF2 plays a role in maintaining mRNA stability [64]. Each YTHDF paralog compensates for the functions of other YTHDF paralogs [60].

YTHDF2 is the first m6A recognition protein to be discovered and has since been studied extensively. It recruits the splicing factor of the precursor mRNA and regulates mRNA splicing and decay. Fang et al. [65] reported that YTHDF2 downregulates LXRα and HIVEP2 via m6A-dependent mRNA attenuation. LXRα plays a crucial role in maintaining the dynamic balance of intracellular cholesterol by regulating cholesterol uptake and excretion, which is vital for glioma proliferation and invasion. Chai et al. [66] demonstrated that YTHDF2 expression was positively correlated with severe malignancy, WHO grade, and poor prognosis of gliomas. Mechanistically, YTHDF2 accelerates UBXN1 mRNA degradation by recognizing METTL3-mediated m6A modification sites on UBXN1 mRNA, activating NF-κB and accelerating tumor progression. Furthermore, YTHDF2 showed GSC-specific dependency and regulates glucose metabolism in GSCs by stabilizing Myc transcripts, thereby promoting GSC growth [67]. Studies have shown that YTHDF1 is upregulated in gliomas and is positively correlated with patient age and tumor grade [68]. At the same time, YTHDF1 is involved in the proliferation and migration of GBM cells mediated by the RNA-binding protein MSI1. It also plays a role in regulating the proliferation, stem cell-like characteristics, and chemotherapeutic resistance in GBM cells [69]. These results suggest that YTHDF1 and YTHDF2 play a role in promoting glioma progression; however, the role of YTHDF3 in glioma has not yet been elucidated.

#### YTHDC1 and YTHDC2

YTHDC1 and YTHDC2 are members of the YTH domain-containing nuclear proteins that regulate RNA splicing and export processes modulated by m6A and are predominantly located in the nucleus. YTHDC1 primarily regulates mRNA splicing and YTHDC2 promotes mRNA degradation. Previous studies have highlighted the pivotal role of YTHDC1 in m6A-mediated alternative splicing [70]. A luciferase reporter assay conducted in HeLa cells revealed that YTHDC2 can boost target translation efficiency by 52% and simultaneously reduce target mRNA abundance by 15% [71].

YTHDC1 reduces the expression of VPS25 and inhibits glioma proliferation through the JAK-STAT signaling pathway [72]. Li et al. [24] discovered that the METTL3-mediated regulation of the splicing factor NMD depends on YTHDC1. The proliferation of U87 cells markedly declined after the overexpression of YTHDC1 and METTL3. In W377A/W428A mutants, the overexpression of METTL3 and YTHDC1 failed to promote the formation of spherical U87 cells, indicating that the ability of YTHDC1 to promote the GBM functional phenotype depends on its m6A-binding activity.

YTHDC2 is a binding protein of the YTH protein family and the only member with ATP-dependent RNA helicase activity. YTHDC2 plays a role in the different methylation levels observed in uterine corpus endometrioid carcinoma, adrenocortical carcinoma (ACC), and endocervical adenocarcinoma (CESC), leading to different prognoses and levels of immune cell infiltration [73]. Furthermore, KM plot analysis has unveiled the prognostic significance of YTHDC2 in the context of LGG [15, 73].

#### IGF2BP1-3

IGF2BPs are mRNA-binding proteins containing a KH domain that can maintain the stability of their target mRNAs and prevent their degradation by binding to the m6A methylation site and acting as a recognition protein [74, 75]. IGF2BPs stabilize numerous mRNA targets aided by cofactors such as HuR, MATR3, and PABPC1, subsequently promoting oncogenic functions in cancers by upregulating the expression of oncogenes such as Myc [76]. Zhan et al. [72] reported that LINC00689 knockdown can inhibit glioma tumorigenesis through the miR-526b-3p/IGF2BP1 axis. Li et al. [77] reported that ubiquitin-like modifications of IGF2BP2 promote glioma VM by regulating the lncRNA OIP5-AS1/miRNA-4950-3p axis. Liu et al. [78] reported that IGF2BP2 is upregulated in glioma microvessels and glioma endothelial cells. Furthermore, they elucidated the intricate regulatory dynamics of IGF2BP2 in the FBXL19-AS1/ZNF765 axis in governing blood-tumor barrier permeability; such insights could be instrumental in enhancing chemotherapy efficacy [78]. In LGG, miR-138 inhibits IGF2BP2 by directly targeting the 3′-UTR of IGF2BP2 mRNA to weaken the EMT process and reduce the invasiveness of LGG [79]. In addition, miRNA-188 has been shown to inhibit human glioma progression by directly targeting IGF2BP2 [80]. CircHIPK promotes glioma progression by regulating the miR-654/IGF2BP3 signaling pathway [81]. EWSR1 promotes glioma progression by cyclizing circNEIL3, thereby blocking HECTD4-mediated ubiquitination, stabilizing IGF2BP3, and promoting glioma progression [82]. miR-4500 inhibits the progression of human glioma by binding to IGF2BP1 [83]. It has been found that SRSF7 promotes the growth of GBM cells by binding to IGF2BP2 [84]. Collectively, these studies suggested that IGF2BPs have a pronounced pro-oncogenic imprint on gliomas, making them potent candidates as therapeutic targets.

#### HNRNPs

The heterogeneous nuclear ribonucleoprotein (HNRNP) family, also known as the m6A methylated binding proteins, includes HNRNPA2B1 (heterogeneous nuclear ribonucleoprotein A2B1), HNRNPC (heterogeneous nuclear ribonucleoprotein C), and HNRNPG (heterogeneous nuclear ribonucleoprotein G), which play important regulatory roles in RNA processing, maturation, and gene expression. hnRNPs have RNA-binding domains that contain RNA recognition motifs, K-homology domains, and arginine/glycine-rich boxes [85]. As a nucleus-localized m6A reader protein, hnRNPA1 is upregulated by EGFRvIII, leading to increased glycolytic gene expression and shorter survival time in GBM. Additional evidence has demonstrated that hnRNPA1 promotes the splicing of the Max transcript and generates Delta Max, which enhances Myc-dependent cell transformation [86]. hnRNPA2/B1 is an oncogene in gliomas; inhibiting its expression leads to the inactivation of the AKT and STAT3 signaling pathways, which inhibit proliferation and enhance apoptosis in U251 glioma cells [87]. Both the IE86 and IE2 proteins of human cytomegalovirus (HCMV) upregulate hnRNPA2/B1 expression, inhibit apoptosis, and promote cell proliferation and migration [88, 89]. β-asarone potentially targets hnRNPA2/B1, inhibiting glioma cell invasion and EMT [90]. Deng et al. [91] demonstrated that HNRNPA2B1 knockdown could reduce the expression of phosphorylated STAT3 and MMP2 and decrease the viability, adhesion, migration, invasion, and TMZ resistance of GBM, which induces apoptosis and reactive oxygen species generation in tumor cells. One study revealed that an increase in hnRNPA2 expression leads to the accumulation of PKM2, indicating the crucial role of hnRNPA2 in increasing cell proliferation and driving GBM progression [92]. HNRNPC has been proven to contribute to tumorigenesis and predict GBM prognosis [93, 94].

#### eIF3

**Fig. 4:**
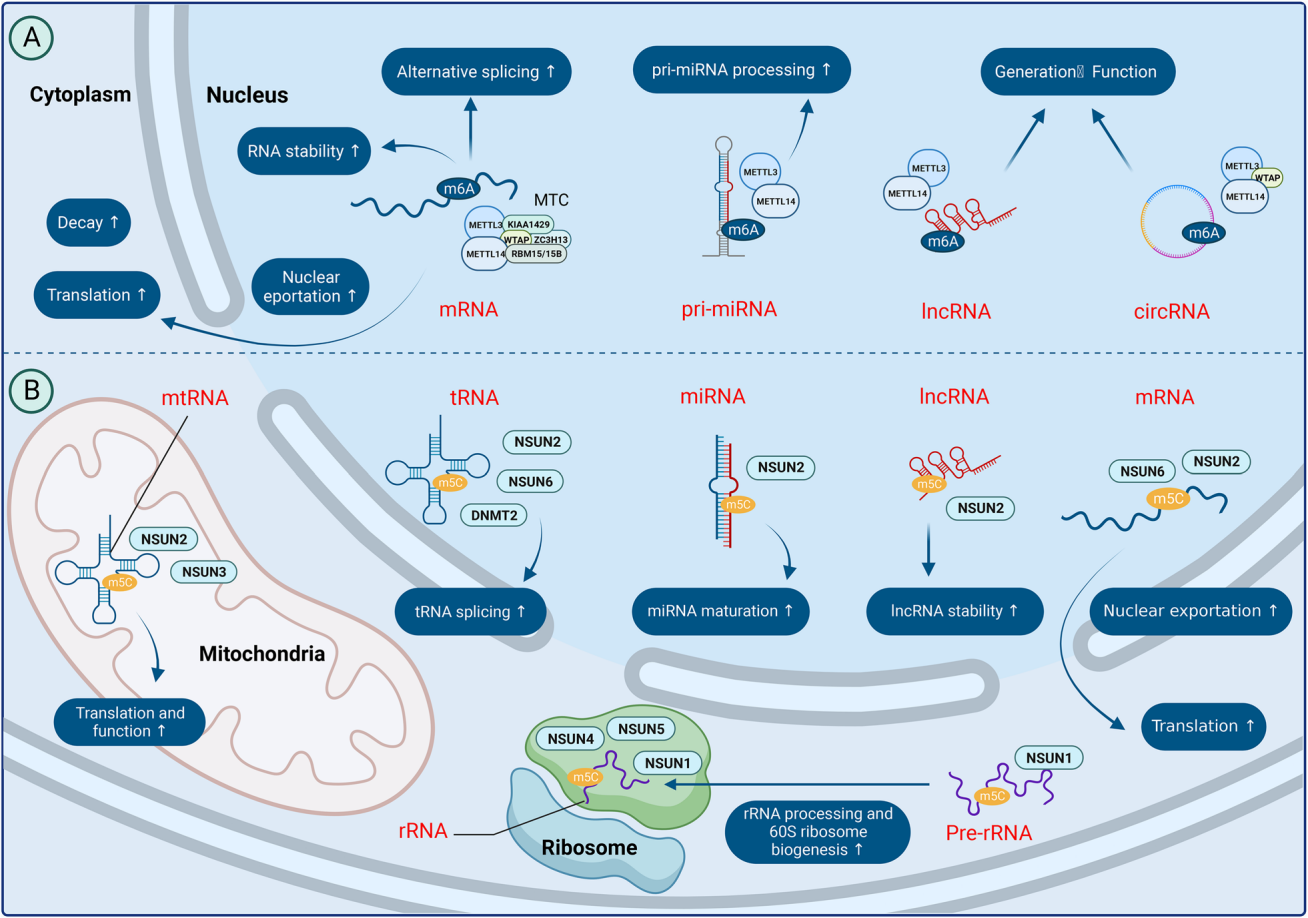
Molecular and biochemical functions of m6A and m5C

Eukaryotic initiation factor 3 (eIF3) can also function as an m6A recognition protein by binding to bases that undergo m6A modification in the 5′-UTR of RNA. This action facilitates mRNA translation and recruits the 43S complex for protein translation in a cap-independent manner [61]. eIF3b knockdown induces G0/G1 phase arrest and apoptosis in U87 cells, significantly inhibiting their proliferation [95]. It has been suggested that eIF3 family members may also play a role in promoting glioma development. The molecular and biochemical functions of m6A are shown in Fig. 4.

#### M5C

##### m5C methylation

m5C RNA methylation refers to the covalent modification of the fifth carbon atom of the cytosine ribose nucleotide in RNA molecules. Initially identified in rRNA in the 1970s, this modification was subsequently observed in tRNAs, mRNAs, and lncRNAs [96–98]. m5C RNA modification is ubiquitous within cells and plays an important role in regulating RNA stability and gene expression.

##### m5C writers

Sadenosylmethionine (SAM) serves as a methyl donor, enabling the m5C methyltransferase to transfer a methyl group to cytosine, yielding 5-methylcytosine [99]. Currently, more than ten RNA m5C methyltransferases have been identified, including the NSUN family, the DNA methyltransferase analog DNMT2, and the tRNA-specific methyltransferase TRDMT family.

##### NSUN family

The human NSUN family consists of seven members: NSUN1–NSUN7, all of which contain the Rossman folding catalytic domain and one SAM-binding site. These proteins catalyze methyl group transfer using a similar mechanism, which involves the formation of a covalent intermediate via covalent binding between the cysteine of the methyltransferase and the cytosine of the RNA. This is followed by the nucleophilic addition of the electron-rich cytosine ring to the methyl group on the SAM to complete methylation. Predominantly found in the nucleus [100]. NSUN1 has been observed to primarily catalyze the m5C methylation of the C2870 site of 25S rRNA in yeast and functionally affect the maturation of 60S rRNA molecule [100]. Recent studies have shown that NSUN1 competitively binds to HIV-1 Tar RNA and catalyzes m5C methylation to inhibit viral DNA transcription [101]. NSUN2, which is encoded on chromosome 10, is a nucleolar RNA methyltransferase that catalyzes various RNA m5C methylation modifications, including those of tRNA, mRNA, and ncRNA [102, 103]. Their roles include cell proliferation, senescence, the cell cycle, epidermal stem cell differentiation, and neural stem cell differentiation [103, 104]. In glioma cells, NSUN2 facilitates m5C methylation to promote the export of ATX mRNA from the nucleus to the cytoplasm in an ALYREF-dependent manner, thereby regulating ATX mRNA expression via methylation and affecting cell migration [105]. NSUN3 is primarily located in the mitochondria and specifically catalyzes the m5C methylation of cytosine at position 34 of mitochondrial tRNA [106]. Both NSUN4 and NSUN5 are rRNA m5C methyltransferases. NSUN4 is located in the mitochondrial 12S rRNA, while NSUN5 is located in the 25S and 28S rRNA [107–109]. NSUN6, a cytoplasmic tRNA methyltransferase, is associated with tRNA shear maturation and is aberrantly expressed in various cancers. Studies have shown that NSUN6 expression is downregulated in testicular, thyroid, liver, and ovarian cancers, and its downregulation often indicates a poor prognosis [110, 111]. NSUN7 is mainly localized within the cellular nuclei and plays an important role in maintaining normal sperm functionality. Mutations in NSUN7 in adult male mice lead to reduced sperm motility and infertility [112].

There are a few reports on the role of the NSUN family in gliomas. Many studies [83] have claimed that NSUN5 deficiency results in a non-methylated state at the C3782 position of the 28S rRNA, strengthening the survival adaptability of glioma cells under stress conditions, potentially worsening the survival and prognosis of patients with glioma.

##### DNMT2

DNMT2 is a tRNA and miRNA methyltransferase mainly located in the nucleus. Unlike the NSUN family of proteins that utilize two catalytic cysteines at the active site, DNMT2 employs a single cysteine at its active site, similar to other DNA methyltransferases [113, 114]. DNMT2 catalyzes the m5C modification of cytosines at position 38 of the tRNA by distinguishing between homologous and near-homologous codons [115]. Elevated DNMT2 activation has been observed in small-cell lung cancer, neuroblastoma, and medulloblastoma [116]. However, the exact mechanism underpinning the role of DNMT2 in glioma remains unknown.

##### M5C erasers

TET1, TET2, and TET3 belong to the ten-eleven translocation (TET) family, representing dioxygenase dependent on Fe (II) and α-ketoglutaric acid (α-KG). Although TET3 is found in both the nucleus and cytoplasm, TET1 and TET2 are primarily located in the nucleus. The TET family was first identified as a family of DNA dioxygenases. Fu et al. [117] found they can also act as RNA demethylases to participate in dynamic RNA cytosine modification. They possess activity against 5-methylcytidine (5mC) and its oxidized derivatives, including 5-hydroxymethylcytidine (5hmC), 5-formylcytidine (5fC), and 5-carboxycytidine (5caC), in both coding and noncoding RNAs [68]. In addition to their roles as RNA demethylases, members of the TET family can also function as DNA demethylases for a variety of nucleic acid substrates, including double-stranded DNA, single-stranded DNA, single-stranded RNA, and DNA-RNA hybridization chains [118].

Diminished DNA repair resulting from TET1 deficiency can lead to genomic instability, which may account for the low survival rates of patients with glioma [119, 120]. In particular, 5hmC is significantly downregulated in gliomas, which may be attributed to a mutation in the isocitrate dehydrogenase genes IDH1/2, resulting in either a shortage of TET or the TET cosubstrate α-ketoglutaric acid. Some studies have suggested that TET3 contributes to the development of GBM by suppressing 5hmC formation.

##### m5C readers

The biological function of RNA modification is primarily related to protein binding. The m5C methylation-binding proteins include YBX1 (Y-box binding protein 1) and Aly/REF export factor (ALYREF).

##### YBX1 (YB-1)

YBX1 is an m5C-binding protein that regulates mRNA stability in the cytoplasm and is involved in the proliferation, differentiation, and malignant transformation of tumor cells [121]. YBX1 exerts pro-cancer effects on glioma progression in multiple ways, regulating the expression and phosphorylation of major proteins associated with the cell cycle, adhesion, and apoptosis [122]. The underlying molecular mechanism in cancer could be attributed to diminished phosphorylation of ErbB, mTOR, HIF-1, cGM-PKG, the insulin signaling pathway, and proteoglycans [123]. Studies have reported that radiotherapy enhances the effects of XVir-N-31-based oncolytic virus therapy using YB-1 in mouse glioma models [2]. YBX1 also acts as an oncogene during tumorigenesis [124]. For example, miR-382-5p has been shown to inhibit the proliferation, migration, invasion, and EMT of glioma cells by targeting YBX1 [125].

##### ALYREF

ALYREF is a key component of the TREX mRNA transporter complex. ALYREF acts as an m5C methylation recognition protein and can specifically bind to m5C-modified mRNA in the nucleus to form mRNP complexes that promote nuclear mRNA export [126, 127]. Recently, it has been found that ALYREF was upregulated in gliomas, suggesting its potential as a prognostic predictor of GBM [128]. The molecular/biochemical functions of m5C are outlined in Fig. 4.

##### M1A

m1A refers to the methylation of the N1 site of adenosine bases in RNA molecules. Abundant m1A modifications have been observed in tRNA and rRNA; however, m1A modification in mRNA is lower, approximately one-sixth of that of m6A [129]. m1A58 methyltransferases (MTases) belong to the RFM superfamily and one of two subfamilies (Trm6 or Trm61). In eukaryotes, the cytosolic m1A58 MTase is composed of a catalytic protein unit from the Trm61 subfamily (Trm61A) and an RNA-binding protein unit from the Trm6 subfamily (Trm6) [130]. The m1A methyltransferase TRMT6 may contribute to glioma progression by modulating the cell cycle and affecting various pathways, including the PI3K-AKT, TGF-β, mTORC1, NOTCH, and MYC pathways [131]. TRM61 functions as a catalytic subunit of the TRM6/61 tRNA methyltransferase. Protein kinase C α (PKCα) interacts with TRM61, regulating the TRM6/61 complex and affecting tumor development by affecting the stability of tRNAi (Met) [132]. TRMT61A is a target of HIF1A. Under hypoxic conditions, TRMT61A levels decrease and suppress c-Myc expression in glioma cells [133].

##### M7G

Currently, METTL1 is the most well-recognized m7G regulator, which binds with its cofactor, WD repeat domain 4 (WDR4), to catalyze m7G modifications in tRNA, miRNA, and mRNA [134]. m7G has a pivotal role in several types of tumors, including gliomas, ovarian cancers, certain sarcomas, breast cancers and so on [9, 135–137]. Li et al. [138] found that METTL1 may promote the malignant behavior of glioma cells through the MAPK signaling pathway. METTL1 expression is elevated in gliomas and correlates with tumor grade [139].

RNA guanine-7 methyltransferase (RNMT) and its cofactor RNMT-activated small protein (RAM) participate in the m7G modification at the 5′ end of mRNA [140]. B7-H6 promotes GSC proliferation via the c-Myc–RNMT axis [141]. rRNA m7G methylation is facilitated by Williams-Beuren syndrome chromosome region 22 (WBSCR22) and tRNA methyltransferase activator subunit 11–2 (TRMT112) [142], while WBSCR22 fosters the glioma cell growth and metastasis by modulating the PI3K/AKT/GSK3β signaling pathway [143].

##### The relationship of RNA methylation between noncoding RNAs and/or canonical RNA-binding proteins

In addition to protein-coding RNAs, noncoding RNAs are also modified in gliomas. Modifications of noncoding RNAs affect the binding of RNA-binding proteins to noncoding RNAs. For miRNAs, HNRNPC directly recognizes and binds to the pri-miR-21 site with m6A modification, subsequently enhancing the expression of miR-21. Therefore, miR-21 promotes cell migration and invasion in glioblastomas [144]. has-mir-346 was found to regulate and bind to the 3′-UTR of YTHDF1 in glioma cells [68]. For lncRNAs and circRNAs, several regulatory mechanisms are involved: m6A modification provides binding sites for m6A reading proteins or regulates the structure of local RNAs to induce RNA-binding proteins to regulate the function of lncRNAs and circRNAs [145]. Moreover, m6A modifications regulate the relationship between lncRNAs and specific DNA sites by affecting the RNA–DNA triple-helix structure [146]. Recent studies highlight that HIF1A-AS2, which is overexpressed in mesenchymal GSCs, negatively affects GSC growth and self-renewal by interacting with the m6A reader IGF2BP2, maintaining the expression of HMGA1. Emerging evidence suggests that circHIPK3 levels increase in gliomas and are correlated with an unfavorable prognosis. By interacting with miR-654, circHIPK3 increases glioma cell proliferation and invasion, thereby stabilizing IGF2BP3.

Canonical non-reader RBPs associated with RNA methylation also interact with modification-containing transcripts without directly recognizing m6A bases. RBPs target adenosine- and uridine-rich elements (AREs) present in the 3′-UTR of RNA. Approximately 8% of human transcription products contain AREs [147] and the regulatory significance of RBPs has been increasing. This usually involves the AUUUA motif, commonly seen in the 3′-UTR of many cytokine- and chemokine-encoding mRNAs, governing RNA degradation and stability.

HuR is a member of the ELAV family of RBPs, known to selectively recognize and bind AREs. It regulates miRNA expression through the AUUUA motif in pri-miRNA. Characterized by its three RNA recognition motif domains, HuR is predominantly located in the 3ʹ-UTR of mRNA near the miRNA binding sites [148]. It was found that the enrichment of RNA methylation is generally at the 3ʹ-UTR end and that HuR can be pulled down by m6A-containing RNAs. Studies have investigated whether the presence of m6A affects HuR binding to RNA; HuR binding reportedly suppresses the inhibitory effect of miRNAs by competing for 3ʹ-UTR binding sites [149]. As exemplified by IGFBP3, a direct target of some miRNAs, HuR promotes mRNA stability by preventing miRNA targeting [150].

Tristetraprolin (TTP), also referred to as ZFP36, is a well-known RBP that regulates mRNA degradation by recognizing AREs in the 3′- UTR of mRNA [151]. This promotes m6A mRNA methylation, which in turn diminishes the stability of CCL2 and CCL5 mRNA. Thus, noncoding RNA and AREs may be potential targets for tumor treatment. By specifically acting on the consensus sequences of noncoding RNA and AU-RBPs, the number of methylated transcripts can be reduced, affecting the expression of downstream genes and regulating the biological function of tumor cells [152, 153].

## Conclusions

Malignant progression and high recurrence rates make gliomas the most lethal primary brain tumors; therefore, understanding the molecular mechanisms of glioma development is imperative to address these challenges. In recent years, RNA modification has emerged as a key area of research owing to its extensive influence on RNA metabolism and function, thus presenting as a promising target for developing novel therapeutic approaches for glioma treatment. Aberrations in epitranscriptomic modification are considered to be one of the key factors driving tumor progression. Among them, methylation modification has the characteristics of being dynamic and reversible, playing an important role in cancer. In this review, we discussed the role of RNA methylation modifications in gliomas and provided evidence from current studies. We suggest that RNA modification writers, erasers, and readers can serve as potential biomarkers for glioma origin, diagnosis, and prognosis, as well as potential drug targets for therapy. Epigenetic regulators such as HDACs, DNMTs, and EZH2 have been confirmed to have significant potential as cancer treatment targets. However, targeting epitranscriptomic factors alone does not always achieve ideal treatment efficacy; it often needs to be combined with other antitumor therapies to achieve optimal results [7, 154].

**Fig. 5:**
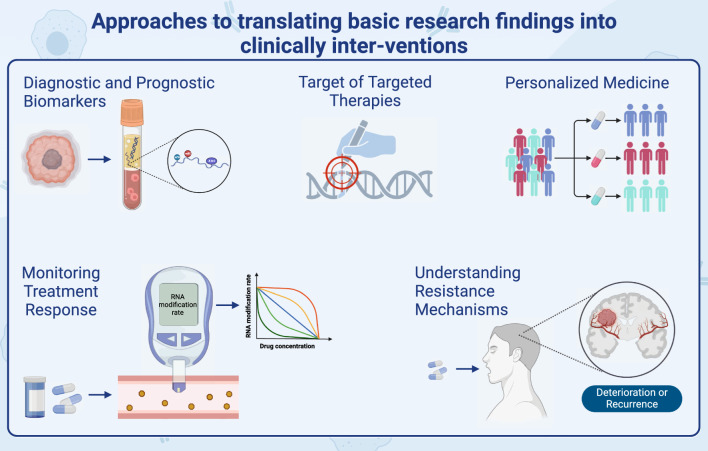
Strategies for translating basic research outcomes into clinical interventions

A multifaceted approach is essential to transition basic research findings into clinically relevant interventions, such as: diagnostic and prognostic biomarkers, targeted therapies, personalized medicine, monitoring treatment response and so on, which are shown in Fig. 5. Concluding, while basic research lays the groundwork, applying these insights to patient care necessitates an intricate strategy that melds drug creation, clinical trials, and a tight-knit partnership between researchers and practitioners. It's vital to comprehend their distinct roles in tumor biology, potential off-target consequences, and the broader ramifications of adjusting methylations in gliomas.

Furthermore, METTL3, FTO, and YTHDC1 were higher in IDH-mutant LGG and GBM than in wild-type gliomas; however, this did not mean that the RNA was more or less methylated (more than m6A/m5C) in IDHm gliomas [36]. Moreover, RNA molecules can have multiple methylation modification sites, and these modifications may act simultaneously. However, determining which modification or regulator plays a dominant role and whether these modifications have synergistic or antagonistic effects can be challenging and may contribute to contradictory findings. Therefore, it is important to study all modifications of this molecule to gain a more comprehensive understanding of their roles in glioma development and progression. Previous next-generation sequencing analyses of RNA have faced limitations, as RNA cannot be directly sequenced. In RNA sequencing, mRNA undergoes fragmentation and is reverse-transcribed into cDNA, which cannot provide full-length transcripts for analysis. However, the advent of third-generation sequencing technologies, notably PacBio and Nanopore technologies, has enabled the accurate identification of multiple homologous isomers of various genes with long reading lengths [155]. These third-generation sequencing technologies can directly identify RNA base modifications and simultaneously detect different types and states of modifications on a molecule, allowing all modifications on a molecule to be considered as a whole while providing high accuracy even at low modification levels. Therefore, this technology has broad application prospects for RNA modifications in epitranscriptomic research.

In summary, this review highlights the potential value of RNA modifications in the diagnosis and treatment of gliomas. Delving deeper into the distinct mechanisms of RNA modification on gliomas is an auspicious future research direction that will refine our understanding of the role of RNA modification in glioma, which may provide more possibilities for the early diagnosis and effective treatment of this life-threatening disease.

## Data Availability

All data generated or analyzed during this study are included in this published article.

## Abbreviations

ARE: Adenosine- and uridine-rich elements
circRNA: Circular RNA
EMT: Epithelial-mesenchymal transition
GBM: Glioblastoma multiforme
GEC: Glioma endothelial cells
GSC: Glioma stem-like cells
LGG: Low-grade glioma
lncRNA: Long noncoding RNA
miRNA: MicroRNA
NMD: Nonsense-mediated mRNA degradation
ROS: Reactive oxygen species
RRM: RNA recognition motif
rRNA: Ribosomal RNA
SCLC: Small-cell lung cancer
SRSF: Serine/arginine-rich splicing factors
TAM: Tumor-associated macrophage
tRNA: Transfer RNA
TET: Ten-eleven translocation
TSS: Transcription start site
UCEC: Uterine corpus endometrioid carcinoma
VM: Vasculogenic mimicry
